# Comprehensive analysis of factors to consider when using glucocorticoids for sCAP: A review

**DOI:** 10.1097/MD.0000000000049670

**Published:** 2026-07-03

**Authors:** Tingrui Zhao, Jing Li, Jing Chen, Jisheng Wang

**Affiliations:** a Department of Clinical Pharmacy, The Third Hospital of Mianyang (Sichuan Mental Health Center), Mianyang, Sichuan, People’s Republic of China; b Department of Neurology, Mianyang people’s Hospital, Mianyang, Sichuan, People’s Republic of China.

**Keywords:** factors, glucocorticoids, hydrocortisone, severe community-acquired pneumonia

## Abstract

Severe community-acquired pneumonia (sCAP) is a serious respiratory disease that often requires urgent and comprehensive treatment. The use of glucocorticoids for the treatment of sCAP has been a focus of discussion in the medical community because of their potential benefits and safety concerns. A literature review showed that the benefits of glucocorticoids in the treatment of sCAP may be related to factors such as oxygenation index, high CRP levels, shock, non-influenza, female gender, early application, and hydrocortisone. The application of glucocorticoids in the treatment of sCAP needs to be considered on an individual basis, weighing the potential benefits and risks, and monitoring and preventing possible side effects.

## 1. Introduction

Severe community-acquired pneumonia (sCAP) is a clinically serious lung infection that poses a threat to patients’ lives and a huge challenge to the healthcare system. This condition is usually caused by bacteria, viruses, or fungi and is characterized by rapidly progressive lung inflammation, which can lead to acute respiratory distress syndrome (ARDS), multiple organ failure, and even death. With the aging of the global population and the increase in immune-compromising diseases, the incidence and mortality rates of sCAP continue to rise, urgently requiring effective treatment strategies.^[1,2]^

The use of antibiotics and antiviral drugs is fundamental for the treatment of sCAP. However, they cannot solve all problems. The inflammatory response is a key link in the pathophysiological process of sCAP, and glucocorticoids (GCs) have become a potential option owing to their potent anti-inflammatory effects. GCs can inhibit the release of inflammatory cytokines and reduce immune cell activation, thereby decreasing the intensity and scope of the inflammatory response.^[3]^

However, the application of GCs therapy to sCAP remains controversial. On the one hand, GCs may alleviate inflammation and improve oxygenation and clinical symptoms;^[4,5]^ on the other hand, they may also suppress the immune response, increase the risk of infection, and delay the clearance of pathogens.^[4]^ Therefore, considering the beneficial factors of GCs in sCAP helps determine the timing of initiating GCs therapy.

In recent years, with an in-depth understanding of the pathophysiological mechanisms of sCAP, as well as progress in clinical and basic research, the application of GCs in sCAP has made new progress. This review aims to explore the relationship between the types of sCAP patients and the benefits of GCs therapy and to look forward to future research directions. By reviewing and analyzing the existing literature, we hope to provide scientific guidance for clinicians to help make more reasonable treatment decisions.

## 2. Materials and methods

A literature search was conducted in PubMed and Embase (2000/01/01–2024/06/30) using MeSH terms for glucocorticoids (including “Glucocorticoids” and “Steroids”) and community-acquired pneumonia. The search was limited to English-language publications.

Inclusion criteria: randomized controlled trials (RCTs), systematic reviews, meta-analyses, or observational cohort studies or reviews; adult patients (≥18 years) with sCAP as defined by ATS/IDSA or other recognized criteria; intervention with any systemic glucocorticoid compared with placebo or standard care; and reporting of clinical outcomes including mortality, need for mechanical ventilation, treatment failure, or adverse events. Exclusion criteria: case reports, case series with <10 patients, editorials, or conference abstracts; pediatric populations; studies focusing exclusively on influenza, COVID-19 without a subgroup for sCAP, or immunocompromised patients; and non-English articles.

Subsequently, the titles, abstracts, and full texts of the retrieved publications were manually reviewed by 2 independent authors (Tingrui Zhao and Jing Li). Disagreements were resolved by discussion with Jing Chen, and the final decision on inclusion was made by Jisheng Wang. Articles were selected for inclusion based on their relevance and content.

### 2.1. The physiological characteristics of sCAP

The hypothalamic–pituitary–adrenal (HPA) axis is dynamically altered across different stages of infection. During the early phase, pathogen-and damage-associated molecular patterns (PAMPs/DAMPs) activate immune cells to release proinflammatory cytokines (e.g., TNF-α, IL-1β, IL-6), which stimulate the HPA axis via neural, humoral, or compromised blood–brain barrier routes, leading to a rapid rise in adrenocorticotropic hormone (ACTH) and cortisol to maintain cardiovascular and metabolic homeostasis.^[6,7]^ This phase is characterized by a sharp rise in ACTH and cortisol secretion, aimed at enhancing cardiac output, maintaining vascular tone, suppressing excessive inflammation, and modulating metabolism, representing an adaptive stress response.^[8,9]^

As infection progresses to the intermediate phase, persistently high levels of inflammatory mediators and oxidative stress may disrupt HPA axis function through hypothalamic/pituitary neuronal apoptosis, reduced ACTH synthesis (partly mediated by nitric oxide), and impaired adrenal steroidogenesis due to lipid droplet depletion and enzymatic inhibition. Concurrently, tissue resistance to glucocorticoids emerges, characterized by downregulation of glucocorticoid receptor (GR) expression, decreased binding affinity, reduced cortisol carriers, and enhanced local inactivation of cortisol to cortisone.^[8,9]^

In the late phase of infection, the HPA axis may become decompensated or exhausted. Although total circulating cortisol levels may remain elevated in some patients, impaired synthesis, tissue resistance, and reduced carrier proteins result in “relative adrenal insufficiency,” clinically manifesting as refractory shock unresponsive to fluids and vasopressors and progressive multiple organ dysfunction syndrome (MODS).^[8,10–12]^

Although it is well-established that GC insufficiency may occur in sCAP, caution must be exercised against excessive exogenous supplementation, as elevated cortisol levels have been associated with increased mortality.^[13]^ Cortisol levels may potentially serve as a prognostic indicator in patients with sCAP.

### 2.2. The action of glucocorticoids

GCs play an important regulatory role in the growth, metabolism, and immune function of the human body. They are the most important regulatory hormones for the body’s stress response and have pharmacological effects such as anti-inflammatory, anti-shock, anti-allergy, and immunosuppressive properties. In terms of anti-inflammatory action, GCs inhibit the dilation of capillaries during the early stages of inflammation, reducing exudation and edema. They also inhibit the infiltration and phagocytosis of white blood cells, lower the permeability of capillaries, and suppress the production of inflammation-related cytokines, thereby alleviating inflammatory symptoms. In the later stages of inflammation, they inhibit the proliferation of capillaries and fibroblasts to effectively delay the formation of granulation tissue, reducing scars and adhesions.^[14-16]^ In terms of anti-shock effects, GCs can inhibit the production of certain inflammatory factors, reduce systemic inflammatory responses, and mitigate tissue damage. They stabilize the lysosomal membrane to decrease the generation of myocardial depressant factors, enhance myocardial contractility, and reduce the sensitivity of blood vessels to certain vasoactive substances, thereby stabilizing microcirculatory hemodynamics.^[17-19]^ GCs exert immunosuppressive effects by inhibiting the phagocytosis and processing of antigens by macrophages, reducing the number of circulating lymphocytes.^[14,16]^ At low doses, they mainly suppress cell-mediated immunity, and at high doses, they inhibit plasma cell and antibody production, thereby suppressing humoral immune function.^[16]^ With their diverse pharmacological actions, there are also various adverse reactions, such as inducing hyperglycemia, hyperlipidemia, increased susceptibility to infections, inducing gastric ulcers, osteoporosis, and an increased risk of thrombosis.^[14,20,21]^

### 2.3. Factors to consider for the benefits of GCs in sCAP

Due to concerns about the quality of RCT data and the differences in the definition of sCAP across various studies, the American Thoracic Society (ATS) and the Infectious Diseases Society of America (IDSA) recommend against the use of GCs therapy in sCAP, except for high-risk patients (asthma, chronic obstructive pulmonary disease [COPD], and sepsis), until more high-quality data become available.^[22]^ In the 2023 European Respiratory Society/European Society of Intensive Care Medicine/European Society of Clinical Microbiology and Infectious Diseases/Latin American Thoracic Association (ERS/ESICM/ESCMID/ALAT) guidelines for the management of sCAP,^[2]^ the recommendations for GCs are cautious, suggesting the use of GCs for patients with septic shock due to sCAP, and not recommending GCs for sCAP without concurrent septic shock. In the 2024 Society of Critical Care Medicine (SCCM) guidelines on the use of GCs in sepsis, acute respiratory distress syndrome (ARDS), and CAP,^[23]^ there is a strong recommendation for the use of GCs in adult patients hospitalized due to severe bacterial CAP, while they are not recommended for patients with mild bacterial CAP. Most guidelines recommend the use of GCs for patients with sCAP accompanied by shock, but there seems to be no consensus on which factors are related to the clinical value of corticosteroid use.

A multicenter, randomized, double-blind, controlled study included 584 patients with sCAP according to ATS/IDSA criteria. No significant difference was found between the methylprednisolone group and the placebo group in the 60-day mortality rate.^[24]^ Similarly, there were no differences in hospital stay, quality of life, or adverse events. However, in a multicenter, double-blind, randomized, controlled study published in the New England Journal of Medicine, it was found that patients who received hydrocortisone had a lower 28-day mortality risk than those treated with placebo, and this advantage could last up to 90 days.^[25]^ The 2 landmark RCTs discussed above – Meduri et al using methylprednisolone (neutral for 60-day mortality) and Dequin et al using hydrocortisone (positive for 28-day mortality) – really highlight how much heterogeneity there is in this field, and clinicians need to be aware of it. Several factors might explain why their results differ so much. For example, timing: in Meduri’s study, 88% of patients got glucocorticoids more than 72 hours after admission, which may have missed the window for calming down the early hyperinflammatory response. In contrast, Dequin’s team started treatment within 15 hours of enrollment. Then there’s treatment duration – Meduri used a 20-day taper, which could have kept patients immunosuppressed for too long during recovery, while Dequin capped therapy at 14 days. The choice of glucocorticoid also matters: methylprednisolone has no mineralocorticoid activity and binds more strongly to the receptor, possibly causing more prolonged immunosuppression than short-acting, lower-potency hydrocortisone. Neither trial used baseline CRP or other biomarkers to select patients, so it’s possible that patients with less inflammation didn’t benefit at all, which would have diluted the effect in Meduri’s study. Finally, disease severity (especially the proportion with septic shock) wasn’t identical between the 2 trials. The take‑home message is that a “one‑size‑fits‑all” approach doesn’t work – the benefit of glucocorticoids in sCAP is highly context‑dependent.

However, it remains challenging to identify which patients with sCAP are most likely to benefit from GCs therapy. The availability of a biomarker or a patient’s clinical status may help optimize the use of glucocorticoids and would be beneficial.^[26]^ In a multicenter study published in JAMA, patients with severe pneumonia and CRP levels > 150 mg/L were included. The study found that the use of GCs could reduce the treatment failure rate in patients with high CRP levels, but there was no significant difference in in-hospital mortality between the high and low C-reactive protein (CRP) groups.^[27]^ The benefits for the highly inflamed population may be related to the significant reduction in CRP and interleukin-6 (IL-6) levels by GCs. Such conclusions cannot be generalized to all patients with CAP, but for patients with sCAP who have a high inflammatory response, the use of GCs may be a beneficial option for this group.

The greater manchester medicines management group (GMMMG) guidelines for Pneumocystis jirovecii pneumonia indicate,^[28]^ “For *Pneumocystis jirovecii* pneumonia, patients are classified as mild-to-moderate or severe based on room air PaO_2_ < 70 mm Hg or an alveolar-arterial (A-a) gradient > 35 mm Hg.” Glucocorticoids are primarily recommended for severe patients, as this classification essentially reflects oxygen supply status (evidence quality was not specified in the guideline). Similarly, for ARDS caused by sCAP, the level of PaO_2_/FiO_2_ is highly correlated with patient mortality rates. The same has been validated in the RECOVERY study, where for COVID-19 pneumonia patients who require mechanical ventilation or have higher oxygen demands, treatment with GCs can reduce mortality. In contrast, for patients with lower oxygen requirements or those who can maintain oxygen saturation with a nasal cannula, the use of GCs does not confer any benefit.^[29]^ The rational use of GCs can reduce the mortality rate in patients with ARDS.^[30]^ A meta-analysis including 10,155 patients with severe or critical COVID-19 pneumonia (defined as tachypnea > 30 breaths per minute, oxygen saturation < 90%, or ARDS) found that a small dose of GCs could reduce the mortality rate of patients. Therefore, according to this study, patients who require oxygen supplementation at a rate of greater than or equal to 10 L/min, noninvasive ventilation, or invasive mechanical ventilation may benefit from GCs therapy.^[31,32]^ Clinicians should pay attention to the trend of changes in the PaO_2_/FiO_2_ level and make rational judgments on the application of GCs, which can provide better conditions for patient survival.

A head-to-head comparison of different GCs is clearly relevant to clinical decision-making. Several research^[33-36]^ converged on a consistent finding: among all glucocorticoids studied, only hydrocortisone was associated with reduced short-term mortality (28- or 30-day) in patients with sCAP, whereas methylprednisolone, dexamethasone, and prednisolone showed no significant mortality benefit. Hydrocortisone also lowered the need for mechanical ventilation, an advantage not observed with the other agents.

Several mechanistic considerations may explain this disparity. Hydrocortisone is a short-acting, low-potency glucocorticoid that possesses both glucocorticoid and mineralocorticoid activity. This dual functionality confers unique therapeutic advantages.^[37]^ The mineralocorticoid effect upregulates angiotensin II receptors, thereby enhancing vasopressor sensitivity and hemodynamic stability – a distinct benefit in sCAP complicated by septic shock – and may also reduce the requirement for vasoactive drugs.^[37]^ In contrast, methylprednisolone and dexamethasone are long-acting, high-potency compounds with pure glucocorticoid activity and no mineralocorticoid effects. Their prolonged receptor occupancy may lead to sustained immunosuppression, potentially impairing pathogen clearance and increasing the risk of secondary infections.^[38]^ Moreover, the shorter half-life of hydrocortisone (8–12 hours) allows for more flexible dosing and rapid withdrawal once clinical improvement is evident, reducing the likelihood of adverse effects such as hyperglycemia and adrenal suppression. Thus, hydrocortisone may modulate inflammation without causing excessive immune suppression – a balance that appears to underlie its clinical benefits in sCAP.

Given the absence of head-to-head randomized controlled trials directly comparing different GCs in sCAP, the current evidence leans toward hydrocortisone, particularly when the therapeutic goals include reducing mortality and preventing progression to mechanical ventilation. Findings from multiple studies suggest that short-term, low-dose GCs therapy is beneficial in sCAP. A regimen of hydrocortisone at a dose not exceeding 400 mg/d for 5 to 7 days is considered reasonable.^[39–41]^ We believe that future trials should specifically randomize patients between hydrocortisone and methylprednisolone to provide definitive guidance for clinical practice. However, these conclusions will require further validation through more rigorous, large-scale, multicenter randomized controlled trials.

## 3. Conclusion

In summary, the decision to use GCs in sCAP requires careful individualization rather than a 1‑size‑fits‑all approach. The current evidence suggests that patients most likely to benefit are those with a high inflammatory response (CRP > 150 mg/L), septic shock, severe hypoxemia (PaO_2_/FiO_2_ ≤ 200 or requiring ≥ 10 L/min of oxygen or non‑invasive/invasive ventilation), and non‑influenza etiology. Among available agents, hydrocortisone appears superior to methylprednisolone, dexamethasone, or prednisolone in reducing mortality and preventing mechanical ventilation, likely due to its short duration of action, lower potency, and mineralocorticoid activity. Early initiation (within 24 hours of admission) and a limited course (≤14 days, typically 5–7 days) are associated with better outcomes and fewer adverse events, though hyperglycemia, fluid retention, and secondary infections warrant close monitoring. Looking forward, high‑priority research includes large‑scale head‑to‑head RCTs comparing hydrocortisone with methylprednisolone, biomarker‑stratified trials (e.g., using CRP, IL‑6, or cortisol levels), optimization of dosing regimens, investigation of long‑term outcomes such as quality of life and adrenal recovery, and cost‑effectiveness analyses across different healthcare systems. Addressing these questions will provide robust evidence to guide clinical practice and improve prognosis for patients with sCAP.

## Acknowledgments

Thanks all group members for their contributions to this review.

## Author contributions

**Conceptualization:** Tingrui Zhao, Jisheng Wang.

**Investigation:** Tingrui Zhao, Jing Li, Jing Chen, Jisheng Wang.

**Resources:** Tingrui Zhao.

**Visualization:** Jisheng Wang.

**Writing – original draft:** Tingrui Zhao, Jisheng Wang.

**Writing – review & editing:** Tingrui Zhao, Jing Li, Jing Chen, Jisheng Wang.
